# Molybdate Reduction to Molybdenum Blue by an Antarctic Bacterium

**DOI:** 10.1155/2013/871941

**Published:** 2013-12-05

**Authors:** S. A. Ahmad, M. Y. Shukor, N. A. Shamaan, W. P. Mac Cormack, M. A. Syed

**Affiliations:** ^1^Department of Biochemistry, Faculty of Biotechnology and Biomolecular Sciences, Universiti Putra Malaysia, 43400 Serdang, Selangor, Malaysia; ^2^Faculty of Medicine and Health Sciences, Universiti Sains Islam Malaysia, 13th Floor, Menara B, Persiaran MPAJ, Jalan Pandan Utama, Pandan Indah, 55100 Kuala Lumpur, Malaysia; ^3^lnstituto Antartico Argentino, Cerrito 1248 (1010), Buenos Aires, Argentina

## Abstract

A molybdenum-reducing bacterium from Antarctica has been isolated. The bacterium converts sodium molybdate or Mo^6+^ to molybdenum blue (Mo-blue). Electron donors such as glucose, sucrose, fructose, and lactose supported molybdate reduction. Ammonium sulphate was the best nitrogen source for molybdate reduction. Optimal conditions for molybdate reduction were between 30 and 50 mM molybdate, between 15 and 20°C, and initial pH between 6.5 and 7.5. The Mo-blue produced had a unique absorption spectrum with a peak maximum at 865 nm and a shoulder at 710 nm. Respiratory inhibitors such as antimycin A, sodium azide, potassium cyanide, and rotenone failed to inhibit the reducing activity. The Mo-reducing enzyme was partially purified using ion exchange and gel filtration chromatography. The partially purified enzyme showed optimal pH and temperature for activity at 6.0 and 20°C, respectively. Metal ions such as cadmium, chromium, copper, silver, lead, and mercury caused more than 95% inhibition of the molybdenum-reducing activity at 0.1 mM. The isolate was tentatively identified as *Pseudomonas *sp. strain DRY1 based on partial 16s rDNA molecular phylogenetic assessment and the Biolog microbial identification system. The characteristics of this strain would make it very useful in bioremediation works in the polar and temperate countries.

## 1. Introduction

Heavy metals pollution is a global problem. Remediation of heavy metals contaminated sites by bacteria is becoming more and more important. Microbes have the amazing ability to resist the toxicity of heavy metals and this property is useful for bioremediation purposes [1–4]. It is very toxic to ruminants and its bioremediation has been reported [5]. Traces of heavy metals due to anthropogenic sources have been found in soils in Antarctic and toxicity effects on fish in the surrounding area have been reported to occur [6–8]. Few studies have been carried out to study the possibility of microbes for the remediation of heavy metals in cold regions. Low temperature microbial reduction of chromium [9] and mercury [10] has been reported.

Molybdenum is a ubiquitous metal and often pollution of this metal comes from mining operations and anthropogenic sources [11]. Molybdenum is fast becoming a global pollutant. Its pollution ranging from several hundreds to thousands of part per million has been documented in water and soils all around the world [11, 12]. It is not toxic to human but deadly to ruminants at several parts per million [13]. Molybdenum deposits have been identified in Antarctica [14] and although these deposits have been banned from mining by the Madrid protocol, the ban is up for review in 2041 [15]. Even without the presence of mining activities, molybdenum has been found as a pollutant in Antarctica due to anthropogenic sources [8]. There is very limited study on the effects of heavy metals to terrestrial Antarctic organisms and precautionary steps in removing heavy metals pollution should be taken.

Bioremediation using microbes may be the only cheap and workable technology to remediate metal contaminants in Antarctica and other cold regions. Thus it is vital that cold-tolerant molybdenum-reducing microbes are isolated from polar regions as a preparative step for future bioremediation works. It is also anticipated that other cold region areas would benefit from this research. In this work we report on the isolation and characterization of a psychrotolerant bacterium with the ability to transform molybdenum to the insoluble molybdenum blue. This is the first molybdenum-reducing bacterium isolated from cold region. This bacterium could be used in future remediation works of molybdenum in Antarctica and cold climate regions.

## 2. Experimental

### 2.1. Isolation of Molybdenum-Reducing Bacterium

Samples were collected from jubany Station, an Argentinean Base on King George Island, South Shetlands Islands, Antarctica (61.5°S 54.55°W) in the austral summer 2002-2003 expedition. Soils were collected 15–20 centimetres (cm) beneath the surface and were placed in sterile screw-capped vials. The samples were immediately placed in a freezer and stored at −20°C until returned to the laboratory for further examination. About five grams of soil sample that was well-mixed were suspended in 45 mL of 0.9% saline solution. A suitable serial dilution of soil suspension was then spread plated onto an agar of low phosphate medium (LPM) (2.8 mM phosphate) (pH 7.0). The medium (w/v) consisted of glucose (1%), MgSO_4_·7H_2_O (0.05%), (NH_4_)_2_SO_4_ (0.3%), NaCl (0.5%), Na_2_MoO_4_·2H_2_O (0.242%), yeast extract (0.05%), and Na_2_HPO_4_·2H_2_O (0.05%) to isolate molybdenum-reducing bacteria [16]. After 72 hours of incubation at 10°C several white and blue colonies appeared. The colony exhibiting the strongest blue intensity was then inoculated into 50 mL of low phosphate medium and incubated at 10°C for 72 hours statically. Incubation under aerobic conditions gave lower amount of Mo-blue compared to static incubation. It was observed that during cellular reduction of molybdate, cells precipitated together with the molybdenum blue product preventing cellular growth. A portion of the growth medium containing Mo-blue was centrifuged at 10,000 ×g for 10 min and the supernatant was scanned from 400 to 980 nm using a Cintra 5 spectrophotometer. A freshly prepared low phosphate medium was used for baseline correction. This bacterium was kept in the Bioremediation and Bioassay Laboratory (Lab 204). The production of molybdenum blue in the culture supernatant was monitored at 865 nm. Identification of the bacterium was performed by using molecular phylogenetics studies and Biolog GN MicroPlate (Biolog, Hayward, CA, USA) according to the manufacturer's instructions.

### 2.2. Optimization of Molybdenum Reduction

Optimization of several parameters that support molybdenum reduction to molybdenum blue such as temperature, pH, the effect of carbon sources as electron donors, the effect of nitrogen sources, sodium molybdate, and phosphate concentrations were studied using the LPM in Section 2.1 as the basal medium for 48 hours statically.

### 2.3. 16s rDNA Gene Sequencing and Phylogenetic Analysis

Extraction of genomic DNA was performed using the alkaline lysis method [17]. PCR amplification was carried out on a Biometra T Gradient PCR (Montreal Biotech Inc., Kirkland, QC, Canada). The PCR mixture consisted of 0.5 pM of each primer, 1x reaction buffer, 2.5 U of Taq DNA polymerase (Promega), and 200 *μ*M of each deoxynucleotide triphosphate, at the final volume of 50 *μ*L. Amplification of the 16s rDNA gene was carried out using the following primers; 5′-AGAGTTTGATCCTGGCTCAG-3′ and 5′-AAGGAGGTGATCCAGCCGCA-3′ corresponding to the forward and reverse primers of 16s rDNA, respectively [18]. PCR was carried out under the following conditions: initial denaturation at 94°C for 3 min; 25 cycles of 94°C for 1 min, 50°C for 1 min, and 72°C for 2 min; a final extension at 72°C for 10 min. Cycle sequencing was subsequently performed with the Big Dye terminator kit (Perkin-Elmer Applied Biosystems) as recommended by the manufacturer. Sequence data were initially recorded and edited using CHROMAS version 1.45. The resultant 1452 bases were compared with the GenBank database using the Blast server at NCBI (http://www.ncbi.nlm.nih.gov/BLAST/). Analysis showed that this sequence to be closely related to *rrs* from Gammaproteobacteria. The 16s rRNA ribosomal gene sequence for this isolate has been deposited in GenBank under the accession number DQ226202.

### 2.4. Phylogenetic Analysis

Twenty-three 16S rRNA gene sequences closely matched to isolate DRY1 were retrieved from GenBank and a multiple alignment of the sequences was carried out using clustal_W [19]. A phylogenetic tree was constructed by using PHYLIP, version 3.573 (J. Q. Felsenstein, PHYLIP—phylogeny inference package, version 3.573, Department of Genetics, University of Washington, Seattle, WA, USA (http://evolution.genetics.washington.edu/phylip.html)) [20], with *Serratia marcescens* as the outgroup in the cladogram. Evolutionary distance matrices for the neighbour-joining/UPGMA method were computed using the DNADIST algorithm program. Phylogenetic tree was inferred by using the neighbour-joining method of Saitou and Nei [21]. With each algorithm, confidence levels for individual branches within the tree were checked by repeating the PHYLIP analysis with 1000 bootstraps by the SEQBOOT program in the PHYLIP package. Majority rule (50%) consensus trees were constructed using the Ml methods [22] and the tree was viewed using TreeView [23].

### 2.5. Crude Enzyme Preparation

Crude enzyme was prepared from a 2 L culture grown at 20°C for 72 hours on an orbital shaker at 150 rpm on a modified high phosphate medium (HPM) consisting of MgSO_4_·7H_2_O (0.5 gL^−1^), (NH_4_)_2_SO_4_ (3 gL^−1^), yeast extract (1 gL^−1^), NaCl (5 gL^−1^), NaMoO_4_·2H_2_O) (12.1 gL^−1^ or 50 mM), glucose (10 gL^−1^) as the source of electron donor, and Na_2_HPO_4_·2H_2_O (100 mM) at pH 7.3. Growth at high phosphate under aerobic conditions prevents Mo-blue production but cells contained high enzyme activity. Experiments were carried out at 4°C unless stated otherwise. Bacterial cells were first harvested at 10 000 ×g for 20 min at 4°C. The pellet was then reconstituted in 15 mL of 50 mM Tris·Cl buffer (pH 7.0) containing 1 mM phenylmethanesulphonylfluoride (PMSF) as a protease inhibitor and 2 mM of DTT. The cells were then sonicated on a Biosonik 111 sonicator on an ice bath and then ultracentrifuged at 105000 ×g for 90 min at 4°C. The supernatant containing the crude enzyme was collected. The enzyme had an optimum temperature at 20°C (data not shown).

### 2.6. Enzyme Assay

Enzyme was assayed at 20°C according to the method of Shukor et al. [24]. The reaction mixture (1 mL) contained 3 mM of 12-molybdophosphate (electron acceptor substrate) in 50 mM citrate phosphate buffer pH 5.0 at room temperature and 100 *μ*L of NADH at the final concentration of 3 mM. Fifty microlitres of enzyme fraction containing about 1 mg protein were added to start the reaction. The absorbance increase in one minute was read at 865 nm. One unit of Mo-reducing activity is defined as the amount of enzyme that produces 1 nmole Mo-blue per minute at 20°C. The specific extinction coefficient for the product; Mo-blue at 865 nm is 16.7 mM^−1^·cm^−1^ [25].

### 2.7. Ion Exchange and Gel Filtration Chromatography

Ammonium sulphate fractionation was omitted as it gave very low enzyme yield. The experiment was carried out at 4°C unless stated otherwise. Crude fraction (cytoplasmic fraction after ultracentrifuge) was directly applied to the strong anion exchange matrix Mono-Q (Amersham Pharmacia). The column was first equilibrated with 50 mL of 50 mM Tris·Cl pH 7.5 (buffer A) containing 0.1 mM DTT. About 40 milligrams of the crude enzyme in 2 mL of volume was injected into the Mono-Q column at a flow rate of 1 mL per minute and then washed with 20 mL of the same buffer. Enzyme was eluted from the column with a linear gradient of 0–0.5 M NaCl in buffer A at a flow rate of 1 mL min^−1^. The protein elution profile was monitored at 280 nm. This process was repeated several times until the entire crude fraction was loaded into the column.

The fractions with enzyme activity were pooled and dialyzed against 5 L of buffer A overnight. The dialyzed enzyme was then concentrated using a cellulose triacetate filter membrane with a molecular weight cut-off point of 10 kDa in an Amicon ultrafiltration cell at 4°C to a final volume of 0.5 mL. One hundred microlitres of sample containing 1.2 mg of protein was applied to Zorbax GFC-250 column (250 × 9.4 mm) and eluted using buffer A containing 0.2 M KCL at a flow rate of 0.5 mL min^−1^. Protein was quantified according to the method of Bradford [26] using BSA as the standard. Michaelis Menten kinetics constants were determined using GraphPad prism nonlinear regression analysis available from http://www.Graphpad.com.

### 2.8. The Effects of Respiratory Inhibitors and Metal Ions

Respiratory inhibitors such as antimycin A, sodium azide, potassium cyanide, and rotenone were dissolved in acetone or deionised water and were added into enzyme assay mixture to the final concentrations of 1.2, 10, 10, and 0.2 mM. Metal ions such as Cr^6+^ (K_2_Cr_2_O_7_, BDH), Fe^3+^ (FeCl_3_·6H_2_O, BDH), Zn^2+^ (ZnCl_2_, BDH), Mg^2+^ (MgCl_2_, BDH), Co^2+^ (CoCl_2_·6H_2_O, BDH), Ni^2+^ (NiCl_2_·6H_2_O, BDH), Cd^2+^ (CdCl_2_·H_2_O, SparkChem), Ag^+^ (AgNO_3_, JT Baker), Mn^2+^ (MnCl_2_·4H_2_O, JT Baker), Cu^2+^ (CuSO_4_·5H_2_O, JT Baker), Hg^2+^ (HgCl_2_, JT Baker), and Pb^2+^ (PbCl_2_, JT Baker) were dissolved in 20 mM Tris·Cl buffer pH 7.0 and were added into enzyme assay mixture to the final concentration of 0.1 mM. Inhibitors and metal ions were then preincubated with 100 *μ*L of enzyme in the reaction mixture at 4°C for 10 minutes minus NADH. The incubation mixture was then warmed to 20°C and 100 *μ*L of NADH at the final concentration of 3 mM was added to start the reaction. Deionised water was added so that the total reaction mixture was 1.0 mL. As a control, 50 *μ*L of acetone was added in the reaction mixture without inhibitors.

### 2.9. Dialysis Tubing Experiment

Briefly, the bacterium was grown in 250 mL high phosphate medium with shaking at 150 rpm at 20°C for 72 hours. Cells were harvested by centrifugation at 15,000 g for 10 minutes. The pellet was washed several times with distilled water and then resuspended in 50 mL of low phosphate solution (pH 7.0) at 20°C containing (w/v) MgSO_4_·7H_2_O (0.05%), NaCl (0.5%), (NH_4_)_2_ SO_4_ (0.3%), yeast extract (0.05%), and Na_2_HPO_4_·2H_2_O (0.05%) with sodium molybdate omitted. About 10 mL of this suspension was transferred into dialysis tubing that was previously boiled for ten minutes (12 kDa molecular weight cutoff). The tube was then immersed in sterile 50 mL of low phosphate medium (pH 7.0) for 10 hours at 20°C statically as described in Section 2.1. Periodically, aliquots (1 mL) of the external medium were taken at the start of the experiment and after an incubation period of 10 hours and then the molybdenum blue produced was monitored at 865 nm. At the same time, 1 mL was taken out from the dialysis tubing and centrifuged at 15,000 g for 10 minutes. The supernatant was then read at 865 nm. Experiments were carried out in triplicate.

### 2.10. Statistical Analysis

Values are means ± SE. All data were analyzed using Graphpad Prism version 3.0 and Graphpad InStat version 3.05 available at www.graphpad.com. Comparison between groups was performed using a Student's *t*-test or a one-way analysis of variance with post hoc analysis by Tukey's test. *P* < 0.05 was considered statistically significant.

## 3. Results

### 3.1. Identification of the Isolate

**Figure 1:**
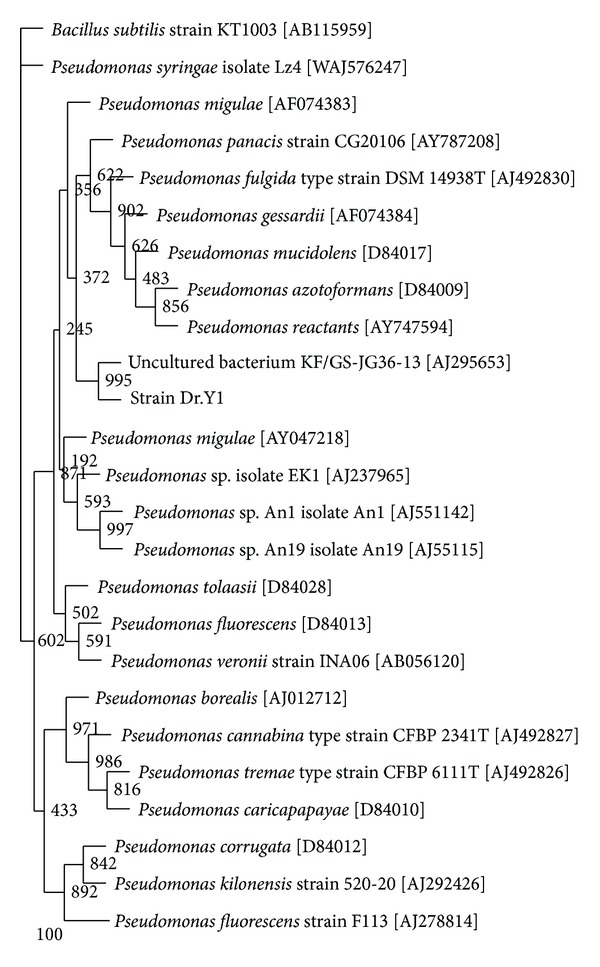
A phylogram (neighbour-joining method) showing genetic relationship between strain DRY1 and other related reference microorganisms based on the 16S rRNA gene sequence analysis. Species names are followed by the accession numbers of their 16S rRNA sequences. The numbers at branching points or nodes refer to bootstrap values, based on 1000 resamplings. Scale bar represents 100 nucleotide substitutions. *B. subtilis *is the outgroup.

A low bootstrap value (<50%) was seen associating isolate DRY1 to several *Pseudomonas* species such as *P. panacis*, *P. fulgida*, *P. gessardii*, *P. mucidolens*, *P. azotoformans*, and* P. reactants *indicating that the tying up of isolated DRY1 to any *Pseudomonas* species cannot be done at this moment. The identifications performed by Biolog GN also gave no conclusive identification to the species level with the closest identification to several *Pseudomonas* species with very low probability. For now, isolate DRY1 is assigned tentatively as *Pseudomonas *sp. strain DRY1 (Figure 1).

### 3.2. The Effect of Electron Donor on Molybdate Reduction

**Figure 2:**
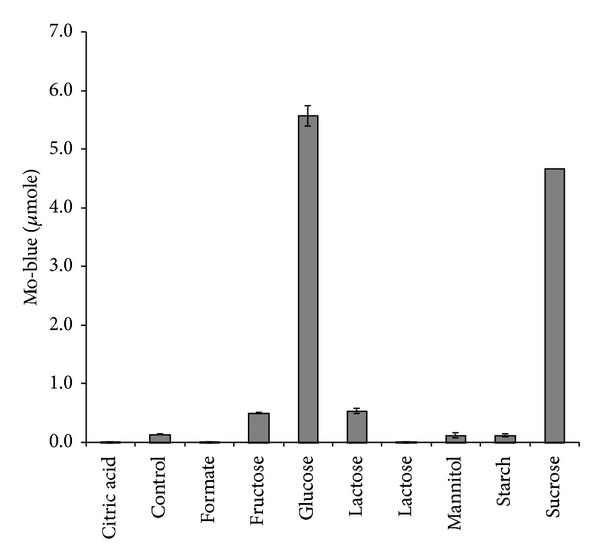
Molybdate reduction using various electron donors. Isolate DRY1 was grown at 10°C for 72 hours in low phosphate liquid medium (pH 7.0) containing (w/v) MgSO_4_·7H_2_O (0.05%), (NH_4_)_2_ SO_4_ (0.3%), NaCl (0.5%), Na_2_MoO_4_·2H_2_O (0.242%), yeast extract (0.05%), and Na_2_HPO_4_·2H_2_O (0.05%) and various electron donors at the final concentration of 0.2%. Molybdate reduction is considered negligible if the absorbance at 865 nm is below 0.020. Error bars represent mean ± standard error (*n* = 3).

The electron donor glucose, sucrose, fructose, and lactose supported molybdate reduction in decreasing order of efficiency with glucose and sucrose exhibiting more than four times the amount of Mo-blue compared to lactose and fructose (Figure 2). Optimum molybdate reduction was achieved at 1% (w/v) glucose after 72 hours (data not shown). The exploitation of the Biolog's carbon sources as electron donors for molybdate reduction was not possible since we discovered that the purplish color of the reduced formazan was too intense and masked the appearance of molybdenum blue. The Biolog system utilizes 95 carbon sources as a metabolic fingerprint for microbial identification.

### 3.3. The Effect of Nitrogen Sources on Molybdate Reduction

**Figure 3:**
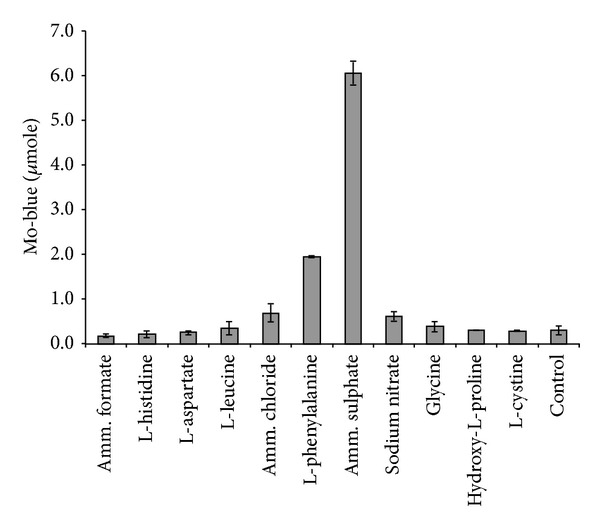
Molybdate reduction using various nitrogen sources. Isolate DRY1 was grown at 10°C for 72 hours in low phosphate liquid medium (pH 7.0) containing (w/v) glucose (1%), MgSO_4_·7H_2_O (0.05%), NaCl (0.5%), Na_2_MoO_4_·2H_2_O (0.242%), yeast extract (0.05%), Na_2_HPO_4_·2H_2_O (0.05%), and various nitrogen sources at the final concentration of 0.2%. Molybdate reduction is considered negligible if the absorbance at 865 nm is below 0.020. Error bars represent the standard error of the mean between three determinations.

The effect of nitrogen sources on molybdate reduction was studied using ammonium formate, ammonium sulphate, ammonium chloride, sodium nitrate, sodium nitrite, oxaloacetate and the amino acids alanine, asparagine, aspartic acid, valine, cysteine, glutamic acid, glycine, histidine, leucine and OH-proline. Ammonium sulphate was found to be the most effective supplement for supporting molybdate (Figure 3). Concentrations of ammonium sulphate giving optimum molybdate reduction were between 0.2% and 0.3%. Further increase in ammonium sulphate concentration shows a strong inhibitory effect on molybdate reduction (data not shown).

### 3.4. The Effect of Temperature and Initial pH on Molybdate Reduction

**Figure 4:**
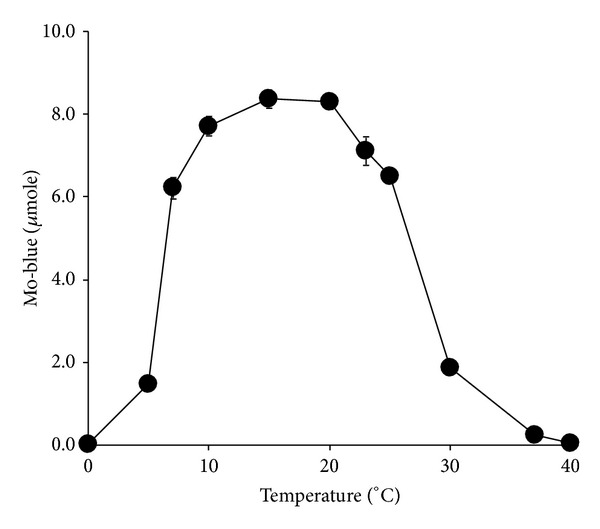
The effect of temperature on molybdate reduction by isolate DRY1. Isolate DRY1 was grown statically at various temperatures for 72 hours in 50 mL low phosphate liquid medium (pH 7.0) containing (w/v) glucose (1%), MgSO_4_·7H_2_O (0.05%), NaCl (0.5%), Na_2_MoO_4_·2H_2_O (0.242%), yeast extract (0.05%), Na_2_HPO_4_·2H_2_O (0.05%), and ammonium sulphate (0.2%). Error bars represent mean ± standard error (*n* = 3).

**Figure 5:**
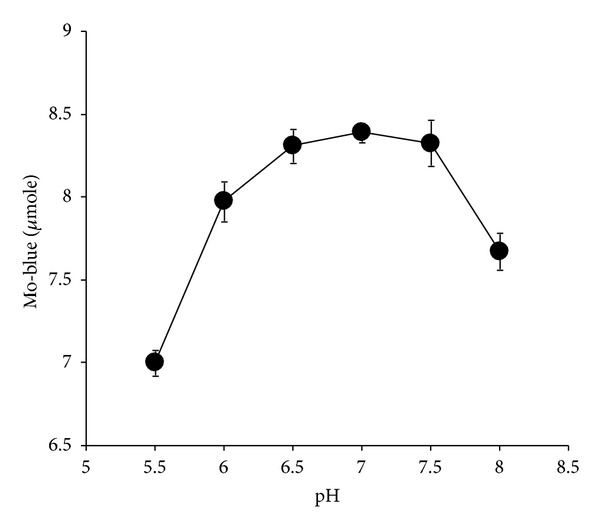
The effect of initial pH on molybdate reduction by isolate DRY1. Isolate DRY1 was grown statically at 20°C for 72 hours in 50 mL low phosphate liquid medium at various pHs containing (w/v) glucose (1%), MgSO_4_·7H_2_O (0.05%), NaCl (0.5%), Na_2_MoO_4_·2H_2_O (0.242%), yeast extract (0.05%), Na_2_HPO_4_·2H_2_O (0.05%), and ammonium sulphate (0.2%). Error bars represent mean ± standard error (*n* = 3).

The effect of temperature on molybdate reduction was carried out at temperatures from 0 to 40°C. The optimum temperature for molybdate reduction was in between 10 and 20°C (Figure 4) with ANOVA analysis showing that there was no significant difference (*P* > 0.05) in terms of molybdate production at these temperatures. The optimum initial pH for reduction was between pH 6.5 and 7.5 (Figure 5) with ANOVA analysis showing that there was no significant difference (*P* > 0.05) in terms of molybdate production at these pHs.

### 3.5. The Effects of Molybdate and Phosphate Concentrations on Molybdate Reduction

**Figure 6:**
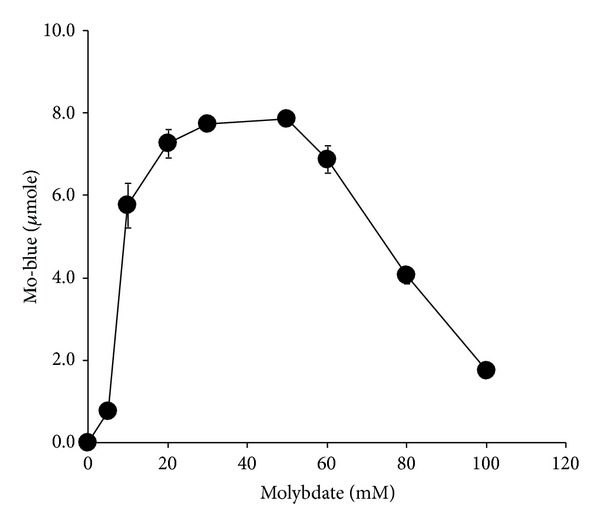
The effects of molybdate concentrations on molybdate reduction by isolate DRY1. Isolate DRY1 was grown at 20°C for 72 hours in low phosphate liquid medium (pH 7.0) containing (w/v) glucose (1%), MgSO_4_·7H_2_O (0.05%), NaCl (0.5%), yeast extract (0.05%), Na_2_HPO_4_·2H_2_O (0.05%), and ammonium sulphate (0.2%). Error bars represent mean ± standard error (*n* = 3).

**Figure 7:**
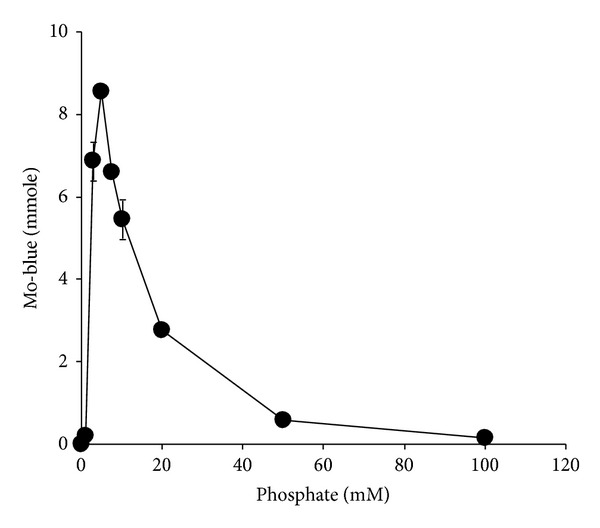
The effects of phosphate concentrations on molybdate reduction by isolate DRY1. Isolate DRY1 was grown statically at 20°C for 72 hours in 50 mL liquid medium (pH 7.0) containing (w/v) glucose (1%), MgSO_4_·7H_2_O (0.05%), NaCl (0.5%), yeast extract (0.05%), ammonium sulphate (0.2%), Na_2_MoO_4_·2H_2_O (1.21%), and various phosphate (Na_2_HPO_4_·2H_2_O) concentrations. Error bars represent mean ± standard error (*n* = 3).

Molybdate reduction was discovered to increase linearly as molybdate concentration was increased from 0 to 40 mM and reached a plateau in between the concentrations of 30 and 50 mM. Concentrations higher than 50 mM were strongly inhibitory (Figure 6). When molybdate concentration was fixed at 45 mM, the optimum concentration of phosphate for molybdate reduction was 5 mM. Molybdate reduction was inhibited at much higher phosphate concentrations and total inhibition occurred at 100 mM phosphate (Figure 7).

### 3.6. Molybdenum Blue Absorption Spectrum

**Figure 8:**
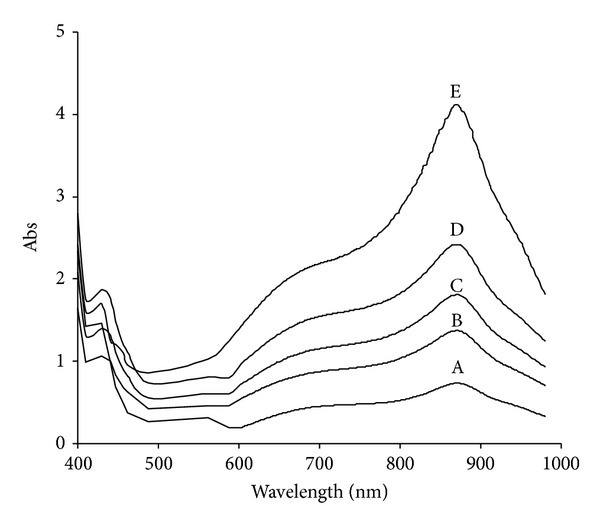
Scanning spectra of molybdenum blue after 28, 32, 36, 48, and 72 hours of static incubation at 20°C labeled (A), (B), (C), (D), and (E), respectively.

The molybdenum blue produced from this isolate showed a spectrum with an absorption profile that exhibited a maximum peak at 865 nm and a shoulder at 710 nm. This unique profile increases proportionately to the blue intensity as molybdate reduction progressed (Figure 8).

### 3.7. Characterization of the Molybdate Reduction Reaction Using the Dialysis Tubing Method

The result showed that 95% of the amount of Mo-blue formed (13.775 ± 0.395 *μ*mole) was found in the dialysis tube while only 5% (1.225 ± 0.025 *μ*mole) was found at the outside of the tube.

### 3.8. Partial Purification of Mo-Reducing Activity

A five-fold purification was achieved after gel filtration but the yield was too low to incorporate another chromatographic step. Native-PAGE results showed that multiple bands indicating purification were not successful (data not shown).  Table 1 shows that ion exchange and gel filtration chromatography removed much of the enzyme yield.

### 3.9. Kinetics of the Mo-Reducing Enzyme

The partially purified enzyme had an optimum pH for activity at 6.0 and optimum temperature for activity at 20°C. Preliminary results show that 20 mM phosphomolybdate (electron acceptor substrate) was saturating. A plot of initial rates against electron donor substrate concentrations at 15 mM phosphomolybdate registered a *V*_max⁡_ for NADH at 26.98 nmole Mo blue/min/mg protein and an apparent *K*_*m*_ of 4.68 mM. The *V*_max⁡_ for NADPH was 29.04 nmole Mo blue/min/mg protein and the apparent *K*_*m*_ for NADPH was 7.39 mM.

At 25 mM NADH (saturating NADH concentration of five times *K*_*m*_), the apparent *V*_max⁡_ and apparent *K*_*m*_ values for phosphomolybdate were 23.48 nmole/min/mg protein and 3.52 mM, respectively. When the electron donor substrate was NADPH, The apparent *V*_max⁡_ and *K*_*m*_ values for phosphomolybdate were 27.75 nmole/min/mg protein and 3.8 mM, respectively.

### 3.10. Effect of Respiratory Inhibitors and Metal Ions on Molybdate Reduction

In this work it was discovered that none of the respiratory inhibitors tested showed any appreciable inhibition of more than 10% to the Mo-reducing activity (Table 2). Metal ions such as chromium, copper, cadmium, lead, silver, and mercury cause more than 95% inhibition of the molybdenum-reducing activity (Table 3). The remaining metal ions tested showed no effect on the molybdenum-reducing activity.

## 4. Discussions

Microbial molybdate reduction to molybdenum blue is an interesting phenomenon and is important not only as a bioremediation tool but also for advancing knowledge on microbial metal reduction. According to Levine [27], molybdate-reducing property was first reported in *E. coli* by Capaldi and Proskauer in 1896 [28]. The first detailed study on this phenomenon was reported by Campbell et al. [29]. This is followed by Sugio et al. [30] and Ghani et al. [16]. From the year 2000 onwards, works on microbial molybdate reduction to molybdenum blue were exclusively carried out on local Malaysian isolates [24, 25, 31–40].

In this work, we report on the first isolation of a Mo-reducing bacterium from Antarctica and a second from the Mo-reducing bacterium from the *Pseudomonas *genera [40]. Further identification of the bacterium to the species level can be done by adding several more polyphasic identification methods such as 16S rRNA gene sequencing, determination of genomic DNA G+C content, DNA-DNA hybridization, and fatty acid profile [41].

Previous works have shown that easily assimilable carbon sources such as sucrose, glucose and fructose are the preferred electron donor source for molybdate reduction. In this work, glucose is the best electron donor or carbon source for supporting molybdate reduction similar to several other Mo-reducing bacteria previously reported [16, 29, 37, 39, 40]. Other Mo-reducing bacteria require sucrose [35, 36, 38]. Ammonium sulphate as the best nitrogen source for supporting molybdate reduction in this bacterium reflects the preference of the bacterium for an easily assimilable nitrogen source and this is also shared by many of the Mo-reducing bacteria isolated so far [16, 31–40]. Since molybdenum reduction is growth associated [16, 31–40], the use of glucose and ammonium sulphate as the carbon and nitrogen sources of choice, respectively, reflect a simple growth-associated process of molybdenum reduction in this bacterium. The requirement of static growth for optimal molybdate reduction may reflects a facultative anaerobic property of this bacterium. Many *Pseudomonas *spp. are obligate aerobes but some have been reported to be facultative anaerobes [42, 43].

The optimum temperature in between 10 and 20°C reflects a psychrotolerant property concurrent with its geographical polar origin. This strain would be very useful in bioremediation works in the polar and temperate countries. Other recently isolated molybdenum reducers exhibit higher optimal reduction in between 30 and 40°C [24, 25, 31–40]. Optimum pH that supported Mo-reduction was within the range of optimum pH of other Mo-reducing bacteria that varies from pH 6 to 8 [24, 25, 31–40].

Both Mo-blue, the detoxification product of molybdate, and molybdate itself are nontoxic with reported production of Mo-blue at 80 mM molybdate in *E. coli*. This bacterium was able to produce comparable Mo-blue at molybdate concentrations similar to other previously isolated strains [24, 25, 31–40]. Furthermore it is able to reduce molybdate to Mo-blue at a high initial concentration of sodium molybdate at 100 mM. Since molybdenum pollution has been reported to reach as high as 2000 ppm (20.8 mM molybdate) [44], tolerance and reduction at concentrations higher than 20 mM are an advantage for a microbe. At this concentration molybdate is lethal to ruminant [45].

Phosphate is a known inhibitor towards Mo-blue production with the mechanism of inhibition involving destabilizing the phosphomolybdate intermediate involved in the reduction of molybdate [24, 25, 31–40]. In this work, the optimal concentration of phosphate supporting molybdate reduction at 5 mM is shared by many Mo-reducing bacteria [24, 31–40]. The optimum ratio of phosphate to molybdate concentrations was similar to the results obtained from *Serratia* sp. strain Dr.Y8 [36], *Enterobacter* sp. strain DrY13 [37], and *S. marcescens *strain Dr.Y9 [38]. This ratio is important since the formation of the proposed intermediate phosphomolybdate in molybdate reduction [31–40] is dependent on exact ratio for optimal formation [46, 47].

The dialysis tubing experiment showed similar result to previous works [35, 39, 40] indicating the prominent role of enzyme(s) in molybdate reduction. The remaining molybdenum blue found in the outside of the tubing is due to a slow diffusion of the molybdenum blue as we discovered in previous works.

Amongst the metal ions, molybdenum is unique in its formation of large heteropoly structure in acidic conditions [47]. We previously suggest the formation of the phosphomolybdate as an intermediate compound based on a unique absorption spectra of the reduced product that shows close similarity to Mo-blue produced in the phosphate determination method using ascorbic acid as the reducing agent [25, 48, 49].

The telltale Mo-blue spectra with a peak increase at 865 nm and a shoulder at 710 nm is a signature spectrum for reduced phosphomolybdate species [48, 49] and is seen in a majority of the Mo-reducing bacteria to date [31–40]. The unique spectrum indicating a common species is involved. Further identification of this species would reveal its structure and once the purified enzyme structure is solved, software such as AutoDock can be used to model the site of the reduction. It is interesting to note that in enzymatic chromate reduction, an intermediate species dichromate with a high negative oxidation state has been observed to dock at the lysine residues and heme centres at the chromate reductase enzyme active site responsible for dichromate stabilization and proton and electron transfer activities. This leads to reduction to the insoluble oxidation state [50]. Due to a similar high negative charge of the phosphomolybdate albeit with a very large structure, the mechanism of reduction could be similar.

The ion exchange stage, though vital as a purification step, removes much of enzyme activity. This was also observed during the purification of the enzyme from EC 48 [33]. Probably, a soluble coenzyme was removed during adsorption to the exchanger or this enzyme is extremely heat-labile in strain DRY1. To date, the enzyme responsible for the Mo-reducing activity in EC 48 or any other bacterium has never been purified. Ariff et al. manage to partially purify Mo-reducing enzyme from the bacterium *Enterobacter cloacae *strain 48 or EC 48 up to the stage of ammonium sulphate fractionation [51]. Further purification using gel filtration on Sephadex G 200 was not successful since no active peak containing Mo-reducing enzyme activity was detected. The apparent *K*_*m*_ for phosphomolybdate, NADH, and NADPH were higher compared to 0.32, 1.65, and 2.13 mM, respectively, for EC 48 [33] suggesting lower affinity of the electron acceptor and donors to the Mo-reducing enzyme from this bacterium compared to EC 48. NADH is the more preferred substrate to NADPH due to the lower apparent *K*_*m*_ values obtained in EC 48 [33] and in this work.

The only known enzyme which could reduce molybdate is molybdate reductase [29]. The enzyme catalyses the reduction of Mo(6+) to the (4+) oxidation state before its integration with the sulphur atoms of a pterin derivative named molybdopterin, cofactor of molybdoenzymes. The purification of the Mo-reducing enzyme would ultimately solve a century-old phenomenon.

The inhibition by toxic metal ions in this study is seen in all of the Mo-reducing bacteria [25, 30–40] and in other heavy-metal reducing bacteria as well [52–54]. The inhibition of metal-reducing activity by heavy metals generally indicates an enzymatic origin and not abiotic [42–44].

In contrast to previous results in EC 48 [16], our results suggest that the electron transport chain (ETC) of this bacterium is not the site of molybdate reduction. In EC 48 the role of the electron transport chain is concluded based on the inhibition of molybdate reduction by cyanide [16]. We recently demonstrated that cyanide causes the reaction mixture to become very alkaline and this was the reason for the inhibition. When the reaction mixture was neutralized upon the addition of cyanide, no inhibition of molybdate reduction to Mo-blue was seen [31]. Similar negative results for cyanide and other respiratory inhibitors have been obtained with recently isolated Mo-reducing bacteria [31–40] confirming that the electron transport chain is not involved in molybdate reduction. The respiratory inhibitors used in this work inhibit certain sites of the ETC. Rotenone is an inhibitor to NADH dehydrogenase whilst sodium azide and cyanide are inhibitors to the terminal cytochrome d oxidase. Antimycin A is an inhibitor to cytochrome b. [55]. The use of respiratory to probe the location or identity of other metal-reducing enzymes in various studies has shown mixed results. Respiratory inhibitors tested in this work such as azide, rotenone, and cyanide had failed to inhibit chromate reduction in *E. coli* [54] and in *Pseudomonas mendocina *[56], while cyanide and azide inhibit the reduction of chromate in *Bacillus subtilis* [53] and oxidation of arsenite in *Alcaligense* sp. [52].

## 5. Conclusion

In conclusion, we have isolated and characterized a molybdenum-reducing bacterium from Antarctic soil. The low optimal temperature requirement for molybdenum reduction confirms the psychrotolerant property of this organism. To our knowledge, this is the first report of an Antarctic bacterium able to reduce molybdate to molybdenum blue. We have studied the effect of nitrogen source and electron donors, temperature, phosphate, and molybdate on the reduction of molybdate to molybdenum blue in this bacterium. Only a partial purification of the enzyme was achieved due to the fact that the low enzyme yield of the final step prevents further addition of a chromatographic step. The similarities and differences seen in this work reflect the unique characteristics of Mo-reducing enzyme from different bacteria. The inability of the respiratory inhibitors to abolish Mo-reducing enzyme activity indicates that the electron transport chain is not the site of reduction. The information obtained in this work is not only important for contributing to the understanding of fundamental mechanism of molybdate reduction but will be extremely important in future works on the bioremediation of molybdenum in polar environments.

## Figures and Tables

**Table 1:** Partial purification scheme of Mo-reducing enzyme from *Pseudomonas  *sp. strain DRY1.

Fraction	Total protein (mg)	Specific activity (Units/mg protein)	Total activity (Units)	Yield %	Fold purification
Crude	400	3	1200	100.0	1.0
Mono Q	36	9.0	324	27	3.0
Zorbax GFC-250	2.4	15.2	36.48	3.04	5.06

**Table 2:** Effect of respiratory inhibitors on molybdate reduction. Data represent mean ± standard error (*n* = 3).

Respiratory inhibitors	% Mo-reducing enzyme activity
Control	100.29 ± 10.13
Cyanide (1.2 mM)	102.41 ± 8.82
Antimycin A (10 mM)	105.71 ± 7.35
Rotenone (10 mM)	91.50 ± 5.81
Azide (0.2 mM)	92.60 ± 10.73

**Table 3:** Effect of metal ions on molybdate reduction. Data represent mean ± standard error (*n* = 3).

Metal ions (0.1 mM)	Molybdenum blue produced (nmole/min/mg)
Control	10.00 ± 0.17
Cr^6+^	0.05 ± 0.02
Fe^3+^	9.44 ± 0.51
Zn^2+^	9.39 ± 0.20
Mg^2+^	9.53 ± 0.23
Co^2+^	9.57 ± 0.21
Ni^2+^	9.35 ± 0.23
Cd^2+^	0.46 ± 0.02
Ag^+^	0.23 ± 0.87
Mn^2+^	9.11 ± 0.21
Cu^2+^	0.00 ± 0.12
Hg^2+^	0.10 ± 0.03
Pb^2+^	0.50 ± 0.11

## Abbreviations

LPM: Low phosphate medium
HPM: High phosphate medium.
